# Machine Learning Prediction of Transthyretin Binding for Thyroid Hormone Transport Disruption for Chemical Risk Assessment

**DOI:** 10.3390/toxics14030240

**Published:** 2026-03-10

**Authors:** Shuaikang Hou, Chao Ji, Christopher M. Reh, Patricia Ruiz

**Affiliations:** 1 Oak Ridge Institute for Science and Education (ORISE), Oak Ridge Associated Universities (ORAU), Oak Ridge, TN 37831, USA; 2 Office of Innovation and Analytics, Agency for Toxic Substances and Disease Registry (ATSDR), Centers for Disease Control and Prevention (CDC), Atlanta, GA 30341, USA; 3 Office of the Director, Agency for Toxic Substances and Disease Registry (ATSDR), Centers for Disease Control and Prevention (CDC), Atlanta, GA 30341, USA

**Keywords:** endocrine-disrupting chemicals, QSAR, thyroid hormone transport, transthyretin binding, Tox24, learning-models, in silico modeling, risk assessment

## Abstract

Endocrine-Disrupting Chemicals (EDCs) disrupt thyroid hormone (TH) synthesis, transport, metabolism, and action, thereby perturbing systemic endocrine homeostasis. Transthyretin (TTR) is a key TH transport protein that regulates circulating hormone distribution and tissue availability, particularly during critical developmental windows. Chemical interference with TTR-binding may alter TH bioavailability and represent a transport-mediated molecular initiating event within thyroid-axis perturbation. Despite widespread exposure, many thyroidal EDCs remain unidentified, and their health effects are difficult to assess due to multiple simultaneous exposures. To support endocrine hazard identification and chemical prioritization within risk assessment frameworks, we developed machine learning-based QSAR models during the Tox24 challenge, using a dataset of 1512 chemicals to predict TTR-binding affinity. Of these, 67% were used for training, 13% for testing, and 20% for validation. Molecular descriptors were selected by first removing highly correlated features and then ranking the remaining descriptors using mutual information regression. The leverage approach was applied to define the models’ applicability domain (AD). Five machine learning algorithms, including gradient boosting regressor (GBR), Random Forest, Lasso Regression, Support Vector Machine (SVM), and regularized SVM models, were developed. The GBR model demonstrated the best overall performance. This model achieved an *R*^2^ of 0.89 on the training set, 0.58 on the test set, and 0.55 on the validation set. The molecular descriptor analysis highlights hydrophobicity, steric effects, branching, connectivity, and ionization/electronic effects as the mechanistic basis for TTR disruption and stabilization, providing structural insight into features associated with thyroid hormone displacement. The AD analysis indicated that 97.5% of the test set and 96.0% of the validation set fell within the reliable descriptor space. Importantly, these predictions extend beyond model benchmarking by informing weight-of-evidence evaluations of thyroid-axis perturbation and supporting the prioritization of chemicals for targeted testing within non-animal new approach methodologies. Overall, this work highlights the application of in silico approaches for screening EDCs, supporting the prioritization and identification of potentially harmful chemicals.

## 1. Introduction

The Tox24 Challenge aims to assess and advance computational methods for predicting the in vitro activity of compounds, building on earlier efforts such as the Tox21 Challenge [1]. This initiative employs advanced computational modeling strategies and artificial intelligence (AI)-based algorithms to develop models that accurately predict how compounds interfere with biological processes solely from their chemical structure data [2,3,4].

The challenge is particularly relevant in the context of endocrine-disrupting chemicals (EDCs). According to internationally accepted definitions (e.g., WHO/IPCS), an endocrine-disrupting chemical is an exogenous substance or mixture that alters function(s) of the endocrine system and consequently causes adverse health effects in an intact organism, its progeny, or (sub)populations. This definition underscores that endocrine disruption requires not only endocrine activity but also evidence of adverse outcomes [5,6,7]. The recent literature further emphasizes that EDCs comprise structurally diverse compounds capable of interfering with multiple endocrine-regulated pathways across the lifespan [8].

The health implications of EDC exposure are broad and extend beyond developmental and reproductive toxicity. Accumulating evidence links EDC exposure to metabolic disorders (including obesity and diabetes), neurodevelopmental alterations, hormone-dependent cancers, cardiovascular dysfunction, and immune dysregulation [9]. Importantly, endocrine disruption is now understood as a systems-level phenomenon involving complex crosstalk among endocrine, metabolic, neurological, and immune pathways rather than isolated receptor-binding events.

Mechanistically, EDCs may mimic, block, or alter hormone functions. While classical models of endocrine disruption have focused on ligand binding to nuclear hormone receptors (e.g., thyroid hormone receptors, estrogen receptors, androgen receptors), emerging evidence demonstrates that EDCs also act through membrane-bound hormone receptors, non-genomic signaling pathways, kinase cascades, epigenetic reprogramming, mitochondrial dysfunction, oxidative stress pathways, and the modulation of hormone synthesis, metabolism, and transport [10]. These diverse molecular initiating events may converge to produce adverse outcomes, reinforcing the need for integrative mechanistic frameworks.

Among the endocrine-sensitive targets increasingly recognized in the literature is the immune system. Multiple lines of evidence demonstrate that EDC exposure can alter innate and adaptive immune responses, promote chronic inflammation, influence autoimmunity, and impair host defense [11,12,13,14,15].

This immunomodulatory dimension is particularly relevant in the context of thyroid hormone biology. Thyroid hormones play critical roles in immune cell differentiation, cytokine production, and immune homeostasis, and bidirectional interactions between the endocrine and immune systems are well established [16,17,18]. Consequently, chemicals that interfere with thyroid hormone transport or signaling may exert downstream immunological effects, further broadening the potential impact of thyroid-disrupting chemicals.

One key protein involved in hormone transport and regulation is transthyretin (TTR), which carries thyroid hormones and retinol (a form of vitamin A) in the bloodstream. Disruption of TTR function by EDCs can lead to significant metabolic and developmental health outcomes, as thyroid hormones are critical for growth and development, particularly during prenatal and early life stages [19,20,21,22,23]. Many TTR-disrupting chemicals act through competitive binding, occupying the T4-binding site on TTR and displacing endogenous thyroid hormones, which alter their transport and bioavailability. Therefore, assessing the potential of various chemicals to bind to TTR is essential for evaluating their endocrine-disrupting potential [24,25,26,27,28,29].

Importantly, TTR-binding represents a transport-mediated mechanism of endocrine disruption rather than a receptor-mediated signaling event. Transport protein interference affects hormone distribution, half-life, and tissue exposure, whereas receptor-mediated activity directly alters transcriptional or non-genomic signaling pathways. Although these mechanisms are sometimes discussed in parallel, they represent biologically distinct molecular initiating events. Clarifying this distinction is essential for appropriately interpreting TTR-binding as an endpoint.

Endocrine disruption often arises from the integration of multiple molecular initiating events across different biological targets. Prior integrative modeling efforts for endocrine-active chemicals—though not exclusively focused on thyroid pathways—have demonstrated that physicochemical properties and molecular interactions can differentially influence transport proteins, nuclear receptors, membrane receptors, and downstream signaling networks. Within this broader endocrine landscape, TTR-binding should therefore be interpreted as one component of thyroid-axis perturbation rather than a stand-alone predictor of thyroid disruption. Predictions of TTR affinity are most informative when considered alongside complementary endpoints, such as thyroid receptor activation, modulation of deiodinase activity, inhibition of hormone synthesis, and immune-related biomarkers.

To address the challenges associated with identifying and evaluating EDCs, innovative computational modeling techniques have gained prominence as a cost-effective and scalable approach. Quantitative Structure–Activity Relationship (QSAR) models and machine learning modeling are at the forefront of this effort [30,31,32,33,34,35]. QSAR models employ statistical methods to correlate chemical structures with biological activities, enabling predictions about how new or untested compounds may behave based on existing data [36,37]. Machine learning modeling leverages large, heterogeneous datasets to identify complex, non-linear structure–activity patterns and improve both prediction performance for biological targets such as TTR-binding affinity [1,2,3,4,38].

The Tox24 Challenge serves as a platform for researchers to apply these computational modeling techniques to predict the binding to TTR [1]. By contributing models and analyses, participants enhance publicly accessible datasets that inform hazard identification and chemical prioritization. Positioned within the broader endocrine and immunotoxicological framework described above, the present work aims to provide a focused contribution on transport-mediated thyroid disruption while acknowledging that comprehensive endocrine risk assessment requires integration across multiple molecular and systems-level endpoints.

The Agency for Toxic Substances and Disease Registry (ATSDR) is dedicated to protecting communities by assessing and mitigating the risks associated with exposure to hazardous substances (https://www.atsdr.cdc.gov, accessed on 3 March 2026). In alignment with this mission, the Tox24 Challenge advances the understanding of chemical toxicity through high-throughput screening technologies and computational models. These approaches enable rapid prioritization of chemicals for further testing, thereby supporting evidence-based public health and early-tier screening and prioritization.

This study aims to (1) develop and validate machine learning-based QSAR models to predict TTR-binding activity using in vitro data from 1512 chemicals provided by the Tox24 Challenge in the U.S. EPA’s ToxCast library, and (2) identify key molecular features that influence TTR-binding affinity within this heterogeneous in vitro dataset, while situating TTR-binding as a transport-mediated molecular initiating event within the broader context of thyroid-axis perturbation and immune–endocrine interactions.

## 2. Materials and Methods

### 2.1. Data Set and Data Representation

#### 2.1.1. Data Set

The TTR-binding activity dataset includes 1512 chemicals from the Tox21/ToxCast program, originating from three library subsets: ph1_v2, ph2, and e1k. These subsets represent the original organizational structure of the ToxCast chemical collection and do not indicate differences in the availability or quality of TTR assay data. The ph1_v2 subset contains 293 chemicals historically enriched for pesticides; ph2 includes 768 additional chemicals; and e1k consists of 799 unique chemicals with known estrogen and androgen receptor active reference chemicals. A total of 1512 chemicals selected from the three ToxCast libraries were subsequently tested by the U.S. EPA using in vitro fluorescent assay at a target concentration of 100 μM. This assay employs the probe 8-anilino-1-napthalenesulfonic acid (ANSA) and human TTR to access binding activity [39]. Chemical activity was calculated against a thyroxine (T4) standard curve, in which the high concentration of T4 represented 100% activity (ANSA completely displaced from TTR) and low-concentration T4 represented 0% activity (ANSA not displaced from TTR) [1,40]. Results are reported as percent activity, calculated as 100 minus the percent of control. It should be noted that negative % activity values, which occasionally arise from natural experimental baseline noise, were retained as is in the dataset without truncation to zero or removal. This approach was chosen to prevent the introduction of artificial bias and to preserve the true distribution of the assay variance for robust machine learning model training. A threshold of 20% activity was selected to define positive binding, as this cutoff is consistently applied across TTR assays and reflects a level of displacement distinguishable from background variability. Thus, the dataset used in this study consists exclusively of in vitro assay data. These datasets were curated and provided through the Tox24 challenge, co-organized with the International Conference on Neural Networks [1,39]. We selected this dataset because (i) the assay provides a high-throughput and well-standardized measure of TTR-binding, (ii) the data are publicly available and widely used for benchmarking predictive toxicology models, and (iii) it offers sufficient sample size and consistency for model development. Recent evaluations have also shown that this dataset provides a diverse and homogeneous chemical foundation suitable for predictive modeling across broad chemical space. This study focuses exclusively on the development and evaluation of machine learning models to predict TTR-binding activity using the curated in vitro assay dataset.

To calculate the molecular descriptors for all datasets, we employed the “Calculate Descriptors” function from the Online Chemical Modeling Environment (OCHEM) database [41], utilizing OEState (2D descriptors), ALogPS (2D descriptor), and Dragon v.7 (3D descriptors) [42,43,44,45]. A total of 1512 molecules were processed, with errors encountered for 13 molecules (11 from the training set, 1 from the testing set, and 1 from the validation test set). These errors were due to a “ValueError: Bad Conformer ID”, indicating that the SMILES strings for these compounds were invalid or could not generate a valid molecular conformer. This resulted in 1499 molecules, each characterized by 5636 descriptors.

As a part of the Tox24 challenge, predefined training, testing, and validation subsets were provided to ensure standardized evaluation across participants. The in vitro dataset utilized in this study comprises molecular structures and descriptors, organized into three distinct subsets: a training set of 1001 compounds (67%), a testing set of 199 compounds (13%) for iterative model refinement, and a blind validation set of 299 compounds (20%) for final evaluation [1]. The model was trained with the training set (1001), evaluated on the test set (199), and validated on the blind validation set (299). Each compound in the datasets is represented by its chemical structure in the Simplified Molecular-Input Line-Entry System (SMILES) format, facilitating the extraction of relevant molecular descriptors [1,2,3,4].

#### 2.1.2. Feature Selection

To enhance the dataset’s quality, we implemented a multi-step feature selection process. Initially, we performed data cleaning by removing variables with missing values and retaining only numerical variables, reducing the number of features from 5636 to 5626. To reduce multicollinearity, we calculated pairwise Pearson correlation coefficients for all descriptors in the training set and constructed an upper-triangle correlation matrix to avoid redundant comparisons. Any descriptor that exhibited an absolute correlation greater than 0.95 with a previously encountered descriptor (based on column order) was removed. This procedure retains the first descriptor in each highly correlated cluster and drops subsequent correlated descriptors, resulting in one representative feature per correlated group. This process further reduced the feature count to 3519. Following this, we applied mutual information regression to rank the importance of each feature in predicting the target variable, using the “mutual_info_regression” function in the “sklearn.feature_selection” python 3.11 module [46,47,48]. We selected the top 0.7% of features with the highest mutual information scores, corresponding to 25 features. The top 0.7% of descriptors ranked by mutual information were retained to substantially reduce dimensionality while preserving the most informative features. This threshold was selected empirically through preliminary experiments evaluating performance across varying retention levels, which indicated a performance plateau beyond approximately 0.7%. Retaining additional descriptors did not materially improve predictive accuracy but increased model complexity and overfitting risk. Accordingly, the selected cutoff reflects a balance between dimensionality reduction, computational efficiency, and performance stability. To ensure that all features were on the same scale, we applied z-score normalization using the “StandardScaler” function from the sklearn.preprocessing module in Python 3.11 to all datasets. This method standardizes features by removing the mean and scaling to unit variance based on the training set. To strictly prevent data leakage, the scaler was fitted exclusively on the training set, and the resulting parameters were then used to transform the test and validation sets. The final 25 features were used as inputs across all machine learning models.

To rigorously evaluate the robustness of the feature selection process against the extreme high-dimensionality of the data, a post hoc bootstrap stability analysis was conducted. The training dataset was resampled with replacement 100 times. In each iteration, the mutual information ranking was repeated across the entire initial pool of 5626 descriptors.

### 2.2. Learning Models

After feature selection and normalization, we built and evaluated five machine learning models on the normalized dataset: Random Forest Regressor (RFR), Lasso Regression (LR), Gradient-Boosting Regressor (GBR), Support Vector Machine (SVM), and Regularized Support Vector Machine (RSVM). These models were internally validated in a 5-fold cross-validation. Each model was trained with GridSearchCV and 5-fold cross-validation to identify the optimal parameters. Model performance was evaluated on training, test, and validation sets.

The RFR combines multiple decision trees with hyperparameters tuned for the number of estimators, maximum depth, and minimum samples required to split nodes. LR, a regularized linear model, was optimized for the alpha parameter, which controls the regularization strength. GBR builds trees sequentially, with tuning focused on the number of estimators, learning rate, and maximum depth. For the SVM, the Support Vector Regressor (SVR) with a Radial Basis Function (RBF) kernel was applied, turning C (penalty), epsilon (error margin), and gamma (kernel coefficient). RSVM extended SVM by testing different kernels (RBF, polynomial, linear), with hyperparameters C, epsilon, and gamma to identify the best kernel–parameter combination. A complete Jupyter notebook containing all code used for model development and analysis is provided in the Supplementary Materials to ensure reproducibility.

### 2.3. Consensus Modeling

The consensus approach combines predictions from multiple machine learning models to leverage their strengths, mitigate their weaknesses, and enhance overall prediction accuracy. We selected the two top-performing models to develop a consensus model. Two methods were used to obtain the consensus model results: (1) weighing by model MSE using Equations (1) and (2) simple averaging using Equation (2).

(1) $$w_{j}=\frac{1}{{MSE}_{j}};\;C_{i}=\frac{\sum_{j=1}^{2}w_{j}\times C_{ij}}{\sum_{j=1}^{2}w_{j}}$$

(2) $$C_{i}=\frac{\sum_{j=1}^{2}C_{ij}}{2}$$

where $C_{ij}$ is the jth model’s prediction for the ith chemical, $C_{i}$ is the consensus prediction for the ith chemical, $w_{j}$ is the weight of jth model, and ${MSE}_{j}$ is the mean squared error of jth model.

### 2.4. Model Performance

For all models, the statistical parameters used to evaluate the performance of these computational models included the coefficient of determination (designated *R*^2^ for training set data, *Q*^2^ for testing and validation set data) and the root mean squared error (*RMSE*). Specifically, *R*^2^ evaluates the internal goodness-of-fit for the training data, whereas *Q*^2^ strictly denotes the predictive performance on the external test and validation sets. The equations are listed below:

(3) $$R^{2}\;or\;Q^{2}=1-\frac{\sum_{i\;=\;1}^{n}{\left(Y_{i}\;-\;\hat{Y_{i}}\right)}^{2}}{\sum_{i\;=\;1}^{n}{\left(Y_{i}\;-\;\frac{1}{n}\sum_{i\;=\;1}^{n}Y_{i}\right)}^{2}},$$

(4) $$RMSE=\sqrt{\frac{1}{n}{\sum_{i=1}^{n}\left(Y_{i}-\hat{Y_{i}}\right)}^{2}\sum_{i=1}^{n}abs(Y_{i}-\hat{Y_{i}})}$$

where *n* is the number of data points, $Y_{i}$ is the i-th observed value, ${\hat{Y}}_{i}$ is the i-th predicted value.

### 2.5. Applicability Domain of the QSAR Model

The Applicability Domain (AD) of a QSAR model defines the chemical space within which the model’s predictions are deemed reliable. In this study, the AD was based on similarity calculations using the “applicable” package in R 4.5.0 [49]. The AD analysis used leverage calculation. A leverage threshold was determined based on the training data, and testing or validation data with a leverage value above this threshold were labeled as out of domain. The leverage value ($h_{i}$) assesses the similarity of a chemical to the descriptor space of the training set and is calculated as follows:

*h_i_* = *x_i_^T^* (*X^T^*·*X*)^−1^·*x_i_* (5)

where *x_i_* is the vector of molecular descriptors for ith compound, *X* is the matrix of descriptors for the training set, and $X^{T}$ is the transpose of the descriptor matrix.

To identify structural outliers, we defined a warning leverage threshold ((h*)) as:

h* = 3(*p* + 1)/*n* (6)

where *p* is the number of descriptors in the model, and *n* is the number of chemicals in the training set.

If *h_i_* ≤ h*, the compound is within the AD, meaning the model can predict its activity reliably. If *h_i_* > h*, the compound is outside the AD, and the prediction for this compound may be unreliable. A Williams Plot is used to visualize the leverage approach for both the test set and the validation set.

### 2.6. Assessment of Individual Prediction Accuracy Using Standardized Residuals

To evaluate the accuracy of individual predictions, we calculated the standardized residuals, which are defined as follows:

(7) $$e_{i}=|Y_{i}-\hat{Y_{i}}|$$

(8) $${\mathrm{Standardized}\;e_{i}=e}_{i}/\sqrt{\frac{\sum_{i=1}^{n}{e_{i}}^{2}}{n-2}}$$

where residual $e_{i}$ is the difference between the actual value ($Y_{i}$) and the predicted value ($\hat{Y_{ᵢ}}$). The sample size is denoted as n. Standardized residuals were calculated by dividing the residuals by their standard deviation.

Here, predictions with standardized residuals of less than 2 (in absolute value) were classified as “good” predictions. In contrast, those with standardized residuals exceeding +2 were labeled as “bad” predictions, indicating potential outliers or inaccuracies in the model’s predictions, consistent with established QSAR validation practices for identifying potential outliers and assessing model reliability [50,51,52].

### 2.7. Visualization and Interpretation of the Machine Learning Models

To evaluate model performance, we applied four data visualization techniques. (1) “Measured vs. Predicted Values” plots were constructed to compare predicted values generated by the models against measured values, enabling a direct assessment of prediction accuracy. (2) A “Residuals vs. Predicted Values” plot was used to examine residual distributions and detect potential model bias or heteroscedasticity. (3) A learning curve plot was generated to evaluate the model’s performance as a function of the training dataset size, providing insights into how the model’s accuracy changes with data. (4) For the two top-performing models, “Feature Importance” analysis was conducted to quantify the relative contributions of individual features to model predictions. These plots provided comprehensive perspectives for assessing predictive accuracy and interpretability.

Although Dragon descriptors enhance predictive performance through comprehensive structural encoding, some are abstract mathematical representations that do not allow direct mechanistic interpretation. Consistent with OECD QSAR Principle 5, model development prioritized predictive robustness for screening-level tool use while feature-importance analysis was used to identify interpretable physicochemical trends where possible, thereby balancing interpretability with performance.

## 3. Results and Discussion

### 3.1. Datasets Comparison and Descriptors Comparison

To evaluate whether the predefined training, test, and validation sets adequately cover the full range of observed TTR-binding values, we calculated summary statistics for the response variable in each subset (Table 1). The three sets show comparable distributions, with similar means (33.44–40.97), medians (21.73–30.60), and standard deviations (33.30–36.46). All subsets span nearly the complete range of TTR-binding values present in the full dataset, with minimum values from −45.0 to −23.54 and maximum values from 110.9 to 111.12. These results indicate that each set is representative of the overall response distribution and that the training, test, and validation partitions collectively capture the full variability of measured TTR-binding.

**Figure 1:**
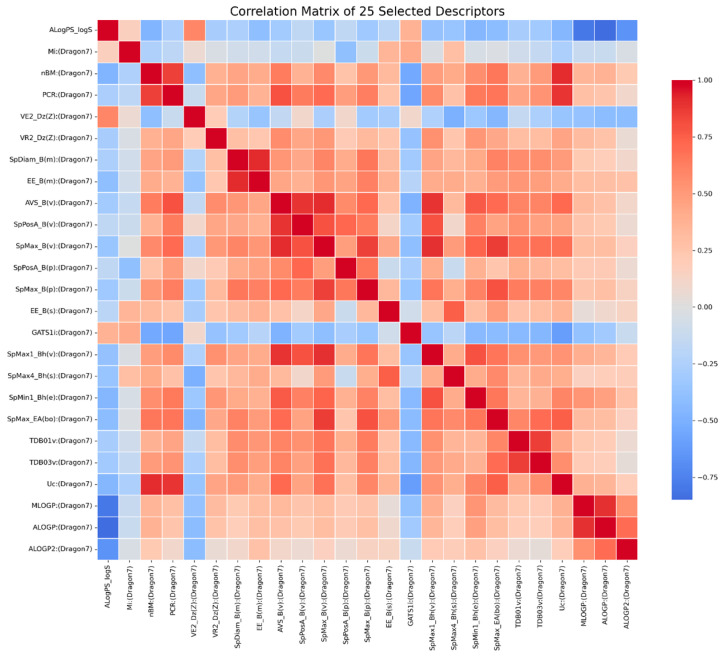
Correlation matrix of the 25 descriptors selected based on mutual information scores.

The resulting 25 descriptors and their pairwise relationships are shown in the 25 × 25 correlation matrix (Figure 1), illustrating the degree of correlation among the selected features. The heatmap displays pairwise Pearson correlation coefficients among the top 0.7% of features with the highest mutual information values (25 descriptors in total). Descriptor names are shown on both axes. The correlation coefficients for these 25 descriptors range from −0.85 to 0.92.

Table 2 summarizes the top 25 molecular descriptors used in model development, including their definitions, descriptor classes, and relevance to structural features and functional groups that influence structural properties and transthyretin (TTR) binding potential.

### 3.2. Model Performance Analysis

The assessment of machine learning models for the Tox24 initiative seeks to elucidate the relationship between molecular properties and the binding affinity of chemicals to TTR [1,2,3,4]. Our developed models and comprehensive analyses have provided significant insights into the correlations between various molecular characteristics and the potential health risks associated with exposure to EDCs and with disruption of thyroid hormone function.

Table 3 summarizes the performance metrics for all evaluated models, including RMSE and $R^{2}$ for the training set, $Q^{2}$ for the test and validation sets, and the cross-validation results. These metrics allow a comprehensive comparison of model performance across all evaluation subsets. The GBR model was the top performer on the test set, surpassing RFR, Lasso Regression, SVM, and RSVM, with the lowest RMSE of 21.47, the highest *Q*^2^ score of 0.58 for the test set, and the highest *Q*^2^ score of 0.55 for the validation set, as shown in Table 2. The similar scores for the test and validation sets indicate that the model maintains consistent prediction accuracy on the validation set, comparable to its performance on the test set. The two consensus models exhibited comparable performance, with identical *R*^2^ and *Q*^2^ values. The “weighing by model MSE” method slightly outperformed than the “averaged” method, as the “Consensus 1” model achieved an RMSE of 21.69 compared to 21.72 for the “Consensus 2” model on the test set, and 11.59 compared to 11.61 on the training set. Overall, both consensus models outperformed the single models, achieving a lower RMSE while maintaining equivalent *R*^2^ and *Q*^2^. This demonstrates a clear correlation between predicted and experimental TTR-binding affinity values. Accordingly, the GBR and consensus models can effectively prioritize chemicals with potential TTR-binding activity based on molecular structures. To more rigorously evaluate model robustness, we performed repeated 5-fold cross-validation using 30 different random seeds and computed the mean RMSE and mean *Q*^2^ across all 150 folds (Table 3). The repeated-CV results show that the average *Q*^2^ values (0.45 for RFR and 0.47for GBR) are lower than the corresponding training *R*^2^ values (0.90 and 0.89), confirming that the models exhibit some degree of overfitting when evaluated on a single train–test split. However, the repeated-CV *Q*^2^ values remain comparable to the external validation *Q*^2^ values (0.55 for both RFR and GBR), indicating stable predictive performance across resampled data partitions. These results suggest that the models generalize reasonably well despite the large difference between training and test *R*^2^, and that repeated cross-validation provides a more realistic estimate of model robustness than a single split.

Although training performance exceeded external validation performance, the models are intended for screening-level tool prioritization rather than quantitative prediction of binding affinity. The Tox24 endpoint represents single-concentration ANSA displacement data and does not provide equilibrium binding parameters; accordingly, the models are designed to rank compounds by relative TTR-binding potential. In this context, moderate reductions in external *Q*^2^ relative to training *R*^2^ are expected given the nonlinear and heterogeneous nature of the dataset. To assess added predictive value, we benchmarked the machine learning models against a baseline linear regression using logP alone. The logP model demonstrated limited performance (Test *R*^2^ = 0.15; RMSE = 30.59), whereas the Random Forest and Gradient Boosting models achieved substantially higher external predictivity (Test *Q*^2^ ≈ 0.54–0.56; RMSE ≈ 22). This improvement indicates that the proposed models capture structural determinants of TTR-binding beyond simple hydrophobicity. Although a gap between training and external performance is observed, consistent cross-validation and independent test results support generalization within the intended screening-level framework. Model outputs should therefore be interpreted as relative ranking tools rather than precise quantitative predictors of binding magnitude.

The performance of the GBR model can be attributed to several factors inherent to its methodology [53]. Firstly, its sequential model-building approach allows each new model to correct the errors of its predecessor, effectively capturing complex patterns within the data. Additionally, the GBR model excels at modeling non-linear relationships, which is particularly advantageous in datasets where such relationships are prevalent, unlike linear models such as Lasso Regression. Furthermore, its flexibility in hyperparameter tuning—such as adjusting the learning rate, the number of trees, and tree depth—enables optimization tailored to specific TTR datasets. Finally, while all machine learning models are susceptible to overfitting, the GBR model incorporates regularization techniques that help mitigate this risk when properly tuned.

The comparatively weaker performance of the non-GBR models can be attributed to several factors. RFR performed moderately well but exhibited greater variance, likely due to sensitivity to descriptor redundancy. LR showed substantially lower performance, indicating that the TTR assay data exhibit a nonlinear structure not captured by a linear model. SVM and RSVM outperformed LR but were less accurate than GBR, consistent with the challenges kernel methods face in very-high-dimensional chemical descriptor spaces. These models highlight potential opportunities for improvement. RFR and SVM-based models may benefit from dimensionality reduction or feature selection to mitigate descriptor redundancy. LR could be enhanced with nonlinear feature expansions, while RSVM performance may improve through kernel optimization or regularization tuning. Future studies could explore these strategies to further strengthen model robustness across algorithmic families.

Although our study focused on traditional machine learning approaches, deep learning provides alternative ways. Results from the Tox 24 challenge [40] demonstrate that deep learning methods—including Transformer-based models, neural networks, and Foundation Chemical Models (MolFormer, SMI_TED and UniMol)—can achieve high predictive accuracy for TTR-binding when appropriately optimized. In the challenge analysis, most of the “group winner” teams successfully applied deep learning or representation learning approaches such as Transformer-CNN, Convolutional Neural Fingerprints, Graph Neural Networks, and fine-tuned Foundation models (e.g., MolFormer, UniMol). These findings suggest that incorporating well-regularized or pretrained deep learning architecture could further improve performance in future work.

**Figure 2:**
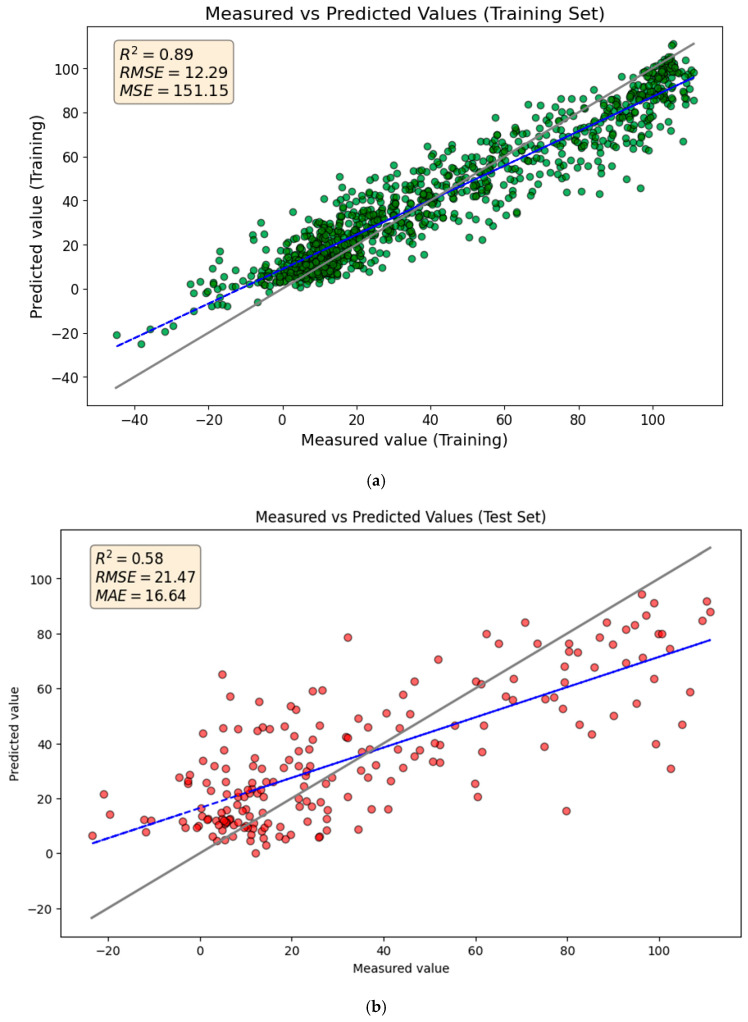
Comparison of measured and predicted values for (**a**) the training set and (**b**) the test set using the GBR model. The solid gray line represents the ideal 1:1 relationship (perfect prediction), while the blue dashed line represents the linear regression fit of the actual predictions.

Figure 2 compares the GBR model’s predicted TTR-binding values with the measured values on the training and test sets. The *R*^2^ and *Q*^2^ scores of 0.89 and 0.58 indicate that the model explains 88% and 56% of the variance for training and test sets, respectively. While the predictions generally follow the trend in the test set data, there are some poor predictions, particularly for higher TTR-binding affinities.

**Figure 3:**
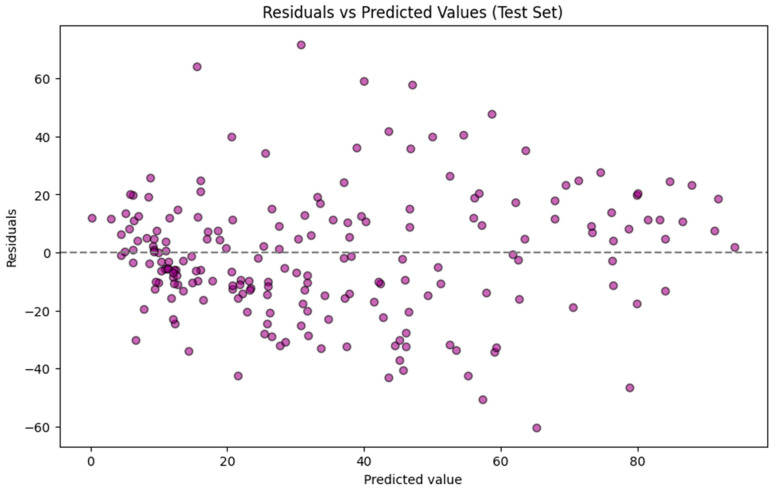
Residuals for predictions of the test set using the Gradient Boosting Regressor model.

The residuals for predictions of the test set by the GBR model are plotted against the predicted values for the test set, as shown in Figure 3. The residuals are primarily centered around zero, indicating no significant bias in the model. However, the spread at higher TTR-binding affinity values reveals some poor performance. Prediction errors are not evenly distributed across the full range of TTR-binding values. A stratified analysis of the testing dataset showed that the mean absolute error for compounds with actual TTR-binding < ~30% (n = 120) was 14.55 while it increased to 26.19 for compounds with actual binding > ~70% (n = 38), indicating a reduced predictive accuracy at higher activity levels. This pattern indicates heteroscedasticity and likely reflects an imbalanced dataset, specifically the underrepresentation of high binders in the training set and the greater structural diversity among strong binders. For practical application, model users should therefore expect higher uncertainty when interpreting predictions above ~30% activity, whereas predictions in the lower range are associated with substantially lower errors.

### 3.3. Applicability Domain Analysis

**Figure 4:**
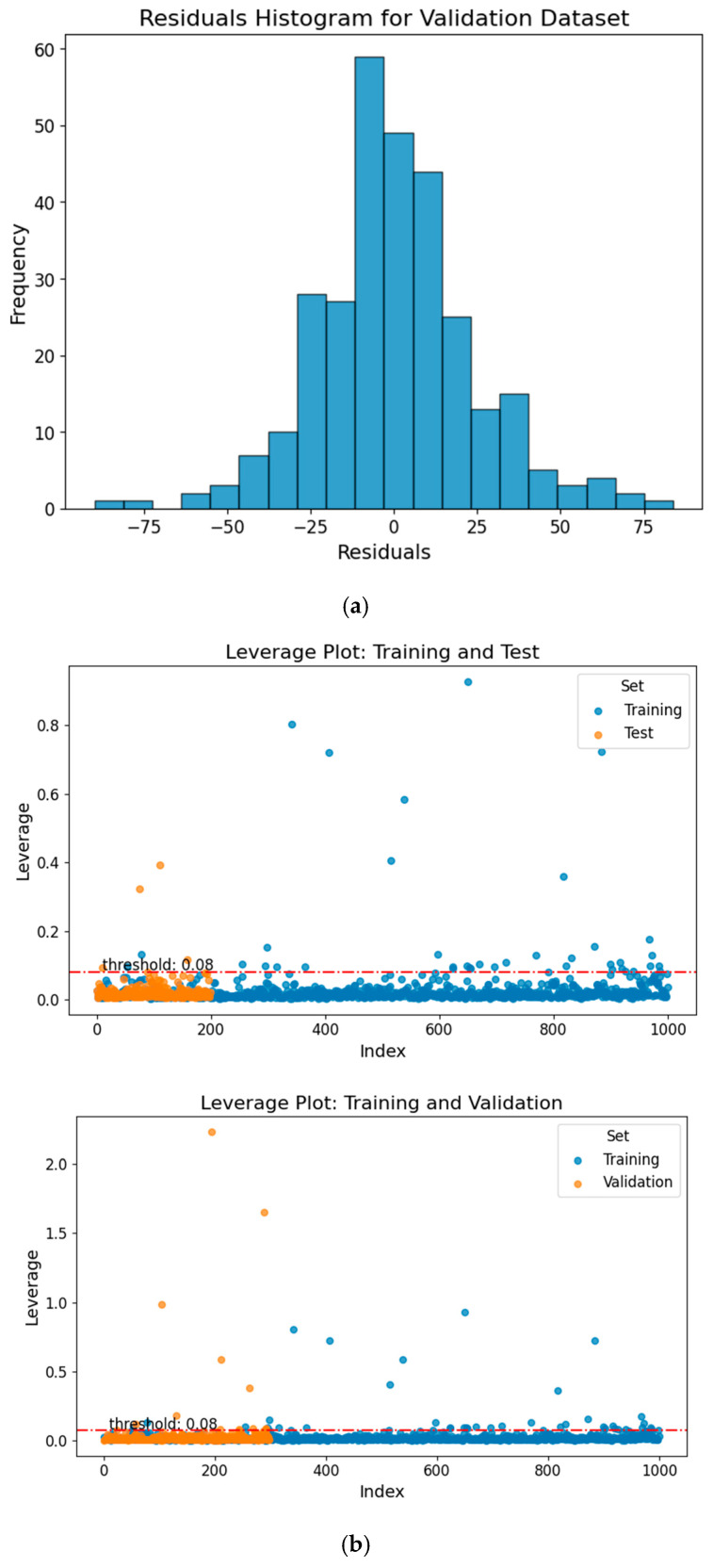
(**a**) Residuals histogram for the validation dataset, illustrating the distribution of prediction errors. (**b**) Williams plot for the test and validation sets, showing model applicability domain and outlier analysis. The red dashed line marks the critical leverage threshold (h* = 0.08). Compounds exceeding this threshold are considered structural outliers.

To evaluate the predictive performance of the machine learning models for each compound, we used standardized residuals as a key metric for assessing their accuracy. Our analysis showed that, among 1499 compounds, the model made 78 “bad” predictions with standardized residuals exceeding 2, corresponding to a “bad” prediction rate of approximately 5.2%. These 78 “bad” predicted compounds tended to exhibit higher hydrophobicity than the overall dataset (higher logP values), with an average logP of 2.94 compared to 2.70 for the full dataset. This suggests that more hydrophobic chemicals may be more likely to produce standardized residuals exceeding 2. These “bad” predictions are mainly due to the poor representation of their chemical classes in the database. We created a histogram of the validation dataset residuals, representing the differences between the actual and predicted TTR-binding affinity values, as shown in Figure 4a. The histogram displayed a normal distribution, suggesting that the prediction errors are symmetrically distributed around zero. This pattern indicates a well-functioning model, as it implies that most predictions fall within 2 standard deviations of the residuals, with a few outliers. Such a distribution of residuals is common in robust predictive models, reinforcing the reliability of the predictions.

A leverage threshold of 0.08 was identified. Chemicals with leverage values greater than this threshold are considered outside the AD, while those with lower values fall within it. The AD analysis results showed that most compounds fell within the reliable descriptor space, with 97.5% of the test set and 96.0% of the validation set falling within this domain, as illustrated in Figure 4b. These findings suggest that the training, test, and validation sets occupy similar regions of chemical descriptor space. Because all subsets were derived from the same chemical inventory, the high percentage primarily reflects internal chemical similarity rather than extrapolation to novel chemical space. Therefore, the AD results should be interpreted as evidence of internal consistency within the modeled dataset, rather than proof of broad external generalizability. The applicability domain (AD) analysis ensures that model predictions are generated within the structural descriptor space represented by the training set. However, inclusion within this descriptor space does not inherently guarantee biological relevance or mechanistic equivalence, particularly for structurally novel or atypical compounds. Accordingly, AD compliance indicates interpolation reliability within the modeled chemical space, rather than predictive certainty in a biological context. Accordingly, predictions for compounds at the boundaries of the descriptor space or possessing unique chemotypes should be interpreted with appropriate caution.

Overall, the results demonstrate the potential of the models for predicting TTR-binding for new EDCs or for chemicals that lack TTR-binding affinity data. This analysis also highlights the need for continuous evaluation of model performance as new data becomes available.

### 3.4. Analysis of Feature Importance

The molecular descriptors were generated from chemical structures, including physicochemical properties such as molecular weight, logP (octanol-water partition coefficient), and polar surface area, as well as topological and electronic features such as molecular connectivity indices and charge distribution. Each molecular descriptor was evaluated for its contribution to the model’s predictive power, allowing us to identify the molecular characteristics that most influence the TTR-binding affinity.

To ensure the reliability of this selection process, a post hoc bootstrap stability analysis (100 resamples) was conducted on the initial pool of 5626 descriptors. Due to the inherent multicollinearity in high-dimensional data, individual selection frequencies were naturally diluted (only six features were selected in >30% of bootstraps). However, 14 of the 25 features (56%) utilized in our final model consistently ranked within the top 100 most frequently selected descriptors. Here, stability refers to the consistency of relative ranking across bootstrap iterations rather than high absolute selection frequency. This pattern indicates that the selected descriptors repeatedly emerged among the most informative predictors, supporting the robustness of the identified predictive domains.

**Figure 5:**
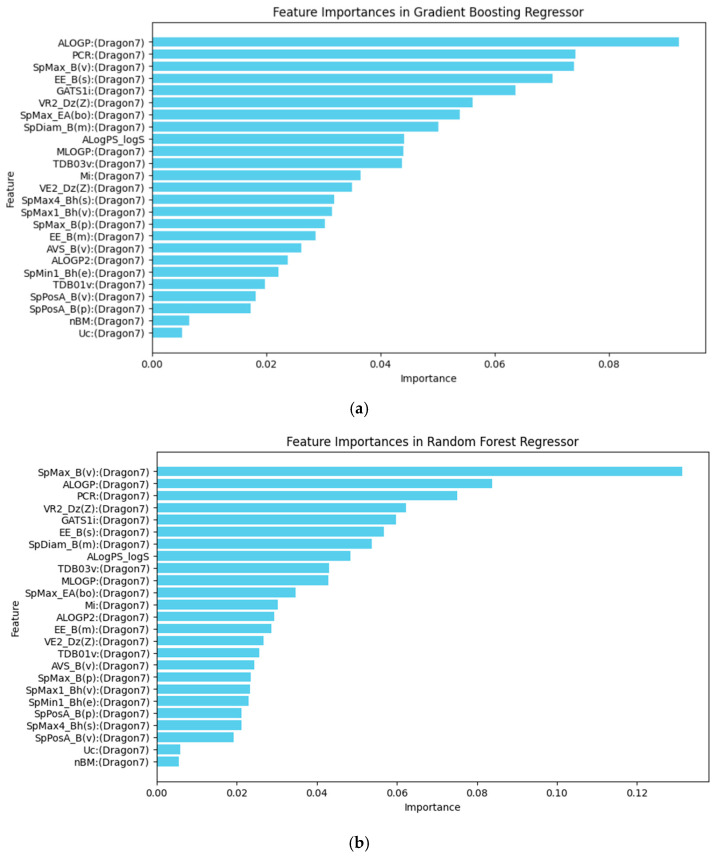
(**a**) Feature importance in the Gradient Boosting Regressor model. (**b**) Feature importance in the Random Forest Regressor model. The horizontal axis represents the relative importance score of each molecular descriptor, which measures its contribution to the model’s predictive power.

In the GBR model, the molecular descriptors/features such as the Ghose–Crippen octanol–water partition coefficient (ALOGP), the leading eigenvalue from Burden matrix weighted by van der Waals volume (SpMax_B(v)) and the ratio of multiple path count over path count (PCR), the Estrada-like index (EE_B(s)), and the Geary autocorrelation of lag 1 weighted by ionization potential (GATS1i) were the most significant contributors to the model’s predictions, as shown in Figure 5a.

**Figure 6:**
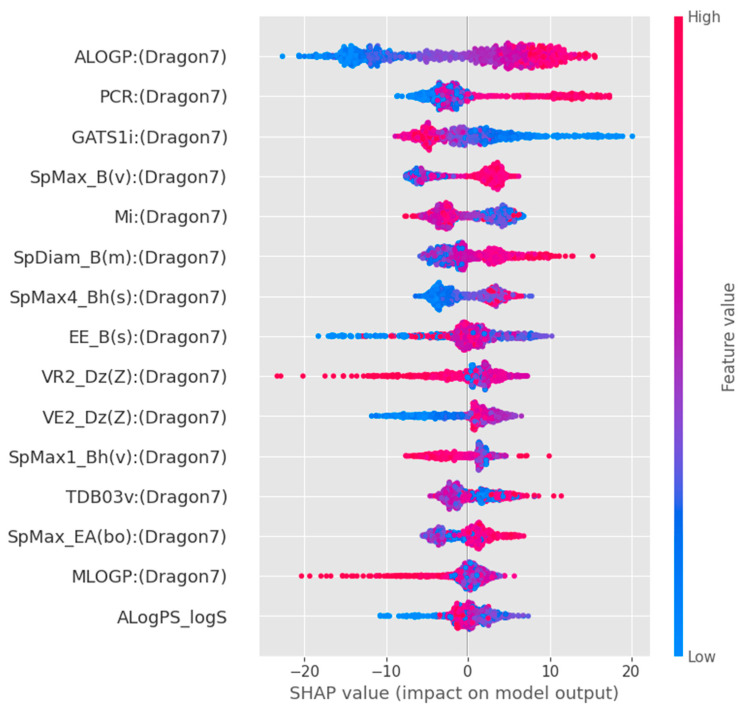
SHAP summary plot for the optimal Gradient Boosting Regressor model. Dot color indicates the feature value (red = high, blue = low), while horizontal position shows the positive (**right**) or negative (**left**) impact on predicted TTR-binding affinity.

To better interpret the directional impact of these key molecular features on TTR-binding affinity, we conducted a SHapley Additive exPlanations (SHAP) analysis on the optimal GBR model (Figure 6). The SHAP summary plot indicates that high values of ALOGP (indicating high lipophilicity) and PCR (structural complexity) are strong and positive contributors to the model’s predicted TTR-binding affinity. Similarly, higher values of SpMax_B(v) (steric bulk) contribute positively to the binding predictions. Conversely, lower values of GATS1i are associated with increased binding affinity. These directional insights indicate that hydrophobicity and steric complementarity are key determinants within the model and are consistent with established TTR-binding interactions. However, these findings reflect model-derived associations rather than direct mechanistic confirmation.

The GBR model results indicated that certain molecular descriptors, particularly those related to hydrophobicity, structural complexity, and electronic properties, were strongly correlated with the observed TTR-binding affinity. For example, compounds with higher octanol–water partition coefficient (ALOGP) values exhibited greater bioaccumulation potential. This trend aligns with cumulative exposure patterns observed in human populations. Additionally, the model highlighted the importance of specific functional groups in mediating toxicity, indicating that compounds containing halogenated aromatic rings, phenolic hydroxyl groups, nitro and aromatic amine functionalities, long hydrophobic or fluorinated chains (including PFAS-like motifs), sulfonyl or sulfonamide groups, and bulky polycyclic scaffolds are more likely to contribute to increased TTR-binding and, consequently, potential adverse health outcomes related to thyroid hormone transport disruption. The TTR-binding site contains hydrophobic regions that facilitate interactions with ligands such as thyroxine and retinol. The ALOGP serves as a measure of a compound’s hydrophobicity, which is critical for its ability to effectively interact with the hydrophobic regions of the TTR-binding site. Compounds with optimal ALOGP values are more likely to exhibit favorable binding affinities, as they can better navigate the hydrophobic environment of the binding pocket, enhancing their interactions with the TTR.

The leading eigenvalue, weighted by van der Waals volume (SpMax_B(v)), serves as an indicator of the steric properties of the ligand. The TTR-binding site has specific spatial constraints that require ligands to possess an appropriate size and shape for optimal binding. The significance of this descriptor in our model indicates that steric compatibility is crucial for ligand binding, as it reflects how well the ligand can occupy the binding pocket without causing steric clashes.

A complex network of interactions, including hydrogen bonds and van der Waals forces, characterizes the TTR-binding site. The ratio of multiple path counts over (PCR) molecular descriptor reflects the complexity and connectivity of the molecular graph, indicating how well a ligand can interact with the binding site. A higher ratio suggests that the ligand can adopt multiple conformations or participate in multiple interactions within the binding pocket, which is crucial for optimizing binding affinity to TTR.

The Estrada-like index (EE_B(s)) offers a comprehensive view of the molecular structure and electronic properties. In the context of TTR-binding affinity, this descriptor captures the overall connectivity and electronic distribution of the ligand, which are essential for effective binding. The TTR-binding site is sensitive to ligand structural features, and including this descriptor in our model emphasizes its role in predicting how well a ligand fits and interacts with the binding site.

The TTR-binding site comprises residues that can be involved in electrostatic and hydrogen-bonding interactions with ligands. The Geary autocorrelation of lag 1 weighted by ionization potential (GATS1i) captures the spatial distribution of electronic properties across the ligand. This descriptor highlights how the electronic characteristics of neighboring atoms can influence TTR-binding interactions. For TTR, ligands that exhibit favorable electronic distributions, as indicated by this descriptor, are likely to form stronger interactions with the receptor, enhancing binding affinity.

The RFR model retains four of the top five same descriptors, including ALOGP, SpMax_B(v), PCR, and GATS1i, as shown in Figure 5b, indicating that these factors are generally important in predicting TTR-binding affinity. However, it substitutes the Estrada-like index with the Randic-like eigenvector-based index from the Barysz matrix weighted by atomic number (VR2_Dz(Z)). This change highlights a different approach to capturing structural complexity, as the Randic-like index focuses on branching and connectivity, weighted by atomic number. This substitution may provide a distinct perspective on how molecular structure influences binding, emphasizing atomic contributions over overall connectivity.

As a note, Dragon descriptors used in the model development include many continuous topological and geometrical indices that, while informative for model fitting, can be difficult to interpret mechanistically because they represent abstract numerical encodings of molecular structure. In contrast, substructure-based descriptors or molecular fingerprints offer more direct structure–activity interpretation and are often preferred when aligning QSAR models with OECD Principle 5 on interpretability. Although the present study focused on Dragon descriptors, we included a detailed analysis of feature importance to provide mechanistic insights in this section. Future work will explore integrating interpretable substructure descriptors to enhance mechanistic transparency and regulatory applicability.

### 3.5. Comparisons of Feature Importance with Existing Studies

Our GBR and RFR molecular descriptors are essential for TTR-binding affinity, consistent with the mechanistic insights—hydrophobicity, steric connectivity, electronic effects, and branching—that have been demonstrated in several studies. Taken together, feature-importance results across models show strong convergence in identifying hydrophobicity, steric bulk, connectivity, and electronic distribution as principal determinants of TTR-binding. Minor differences reflect algorithm-specific weighting rather than divergent biological interpretation. These include studies across halogenated phenols and thiophenols [54,55], Polyfluoroalkyl chemicals (PFCs) [54,55,56,57,58,59,60,61], Polybrominated diphenyl ethers (PBDEs) and Brominated Flame Retardants (BFRs) [62,63], dust chemicals metabolites [64,65], and potent Transthyretin Kinetic/Amyloidogenesis Inhibitors [38]. Collectively, these studies employ diverse modeling and experimental approaches, including single-descriptor QSARs, 2D and 3D QSAR models, and integrated frameworks combining ligand-based descriptors, docking, metabolic simulation, and machine learning. Together, they provide strong support for predictive modeling of TTR-binding chemicals [54,57,58,59,60,66,67,68]. This shows that our models and molecular descriptors are not only statistically important but also mechanistically meaningful.

Feature-importance analysis identified hydrophobicity, steric complementarity, and electronic descriptors as primary contributors to predicted TTR-binding. While consistent with prior structural and competitive binding studies of TTR [61,69], these molecular determinants also represent well-established drivers of endocrine-disrupting activity across multiple biological targets. Lipophilicity and ionization state influence membrane permeability, bioaccumulation, and protein–ligand interactions, thereby shaping both transport protein binding and nuclear receptor engagement [70,71]. Importantly, structural features associated with TTR displacement frequently overlap with those governing thyroid hormone receptor activity and other endocrine-relevant pathways [6,72]. Thus, TTR-binding should not be considered an isolated molecular endpoint but rather a component of a broader endocrine perturbation network that affects hormone distribution, receptor activation, and downstream physiological responses [73]. Situating TTR-binding within this integrative framework enhances the relevance of in silico prediction tools for cumulative and pathway-based endocrine hazard assessment.

Specifically, studies on halogenated phenols and thiophenols consistently demonstrate the importance of ionization state for TTR-binding. The anionic forms bind more strongly than their neutral counterparts due to electrostatic interactions and cation–π contacts with Lys15, while neutral species depend primarily on van der Waals forces [55,74]. Molecular descriptors associated with hydrophobicity, ionization potential, and electronic distribution are therefore highly relevant. This agrees with our models, which identified ALOGP and GATS1i as significant predictors, indicating that the balance of lipophilicity and electronic charge distribution explains a significant portion of the variability in binding across phenolic compounds.

For PFCs, chain length, hydrophobicity, and anionic substituents, such as sulfonates and carboxylates, drive binding affinity [54,56,57,58,59,60,61]. Molecular docking and dynamics confirm that anionic PFCs orient their charged headgroups toward the TTR-binding channel, optimizing electrostatic complementarity and hydrogen bonding [54,61]. QSAR models consistently identified hydrophobicity descriptors and electrostatic/charge-related descriptors as key features [57,59,60]. Our models highlight ALOGP and GATS1i as top predictors, in agreement with this mechanistic evidence. Furthermore, the inclusion of steric and volume descriptors (SpMax_B(v)) in our models aligns with studies that showed larger, bulkier PFAS interact favorably if sterically complementary to the binding pocket [58]. The good predictive performance of our 2D matrix-based indices (EE_B(s), VR2_Dz(Z)) also agrees with published QSAR work emphasizing connectivity and substitution effects as determinants of TTR-binding affinity [59,60].

Yang et al. reported that hydroxylated Polybrominated Diphenyl Ethers (PBDEs) (HO-PBDEs) and related brominated flame retardants show binding dominated by hydrogen bonding interactions with Asp74, Ala29, and Asn27, with Asp74 identified as especially important [63]. Substitution patterns are crucial: ortho-bromination, combined with unsubstituted meta and para positions, enhances both TTR competition and androgen antagonism, although it also correlates with reduced metabolic stability [62]. In this context, our model’s inclusion of PCR is noteworthy, as it captures substitution and branching complexity that strongly influences the structure–activity relationship in PBDEs. Likewise, SpMax_B(v) provides a measure of steric complementarity, which is essential for differentiating strongly versus weakly binding congeners. Thus, our model descriptors provide a mechanistic bridge to explain the steric and branching effects that have been long documented in PBDEs/BFRs structure–activity studies.

Metabolic transformation of environmental contaminants often increases TTR-binding affinity [64,65]. Hydroxylated and halogenated metabolites were predicted and experimentally validated to bind more strongly than their parent compounds, highlighting the importance of bioactivation [64,65]. Molecular docking identified Ser117A as a key residue stabilizing binding across multiple dust-derived ligands. Our models’ identification of molecular descriptors such as ALOGP and GATS1i supports these findings; hydroxylation changes both lipophilicity and electronic distribution, which are precisely the types of molecular changes our model features identify. Additionally, topological descriptors such as PCR and EE_B(s)/VR2_Dz(Z) in our models reflect the importance of connectivity and substitution patterns in hydroxylated metabolites identified in previous QSAR studies.

For therapeutic stabilizers, such as Transthyretin Kinetic/Amyloidogenesis Inhibitors, models like the Predicted Efficacy Score (PES) and the refined Thyroid Hormone Receptor Experimental Efficacy (THREE) Score have been developed to balance efficacy against off-target thyroid receptor binding [38]. These studies identified substructural features and hydrophobic/aromatic complementarity as critical, while docking confirmed that a binding energy “sweet spot” between −7.5 and −9.0 kcal/mol corresponds to selective and potent stabilization [38]. Our molecular descriptors ALOGP and SpMax_B(v) directly capture these hydrophobic and steric features, while EE_B(s)/VR2_Dz(Z) reflect the compactness of stabilizing scaffolds. This shows strong mechanistic agreement that therapeutic inhibitors, such as Amyloidogenesis Inhibitors and environmental disruptors, share lipophilic, steric, and connectivity molecular properties of TTR-binding.

### 3.6. Challenges and Future Directions

Our study successfully developed machine learning models to predict how chemicals bind to TTR; however, some limitations must be acknowledged. First, the modeled endpoint represents percent ANSA displacement measured at a single concentration (100 µM). As a single-point fluorescence displacement assay, this measurement does not provide full concentration–response characterization or binding affinity estimates. Non-monotonic responses, saturation effects, or concentration-dependent artifacts may occur at higher concentrations. Additionally, fluorescence-based assays can be susceptible to compound autofluorescence, quenching, or inner-filter effects, which may introduce variability unrelated to true target engagement. Potential cytotoxicity or nonspecific interactions at 100 µM may also contribute to assay-level noise. Because the dataset was derived from published screening results, detailed replicate-variability metrics were unavailable. Assay variability may therefore impose an upper limit on achievable model performance. Future work incorporating dose–response data and replicate-level uncertainty estimates could further improve predictive modeling of this endpoint.

The Tox24 TTR-binding endpoint is based on single-concentration ANSA displacement, enabling high-throughput screening but imposing biological constraints. Because activity is measured at a fixed concentration, the assay does not capture concentration–response behavior, equilibrium binding parameters, competitive dynamics with endogenous thyroxine (T4), or distinctions between specific and non-specific displacement. Consequently, predicted displacement percentages cannot be directly equated with quantitative perturbation of thyroid hormone transport. To evaluate whether these endpoint characteristics influence model behavior, we conducted a stratified analysis across empirically defined activity ranges (<30%, 30–70%, >70% displacement). Model residuals were centered near zero for intermediate binders (n = 356), whereas systematic shifts emerged at the extremes: low binders (n = 764) showed negative residuals, and high binders (n = 379) exhibited positive residuals with increased dispersion. The elevated uncertainty among highly active compounds is consistent with both activity-space imbalance and assay-related signal compression near saturation. Together, these findings suggest that reduced predictive accuracy at high displacement levels reflects intrinsic assay limitations and dataset imbalance, rather than model inadequacy alone. Importantly, they underscore that, while the model captures relative binding trends, the translation of predicted values into biologically meaningful perturbations of thyroid hormone transport requires cautious interpretation and, ideally, complementary concentration–response or mechanistic validation.

Additionally, the training dataset may not fully capture the diverse chemical space, potentially limiting the model’s ability to predict TTR-binding affinity for new compounds. Moreover, the performance gap between the training set (*R*^2^ = 0.89) and the test set (*R*^2^ = 0.58) suggests potential overfitting, underscoring the need for stronger data regularization and model improvements. Furthermore, the selection of molecular descriptors (25 descriptors in the final model) might overlook important features that could enhance model performance. Incorporating additional mechanistic features, such as quantum-mechanical descriptors, could be considered in future work to further improve model robustness. The complex nature of chemical interactions with the TTR-binding site suggests that exploring additional descriptors, such as those that capture dynamic changes or interaction effects, could yield valuable insights. Moreover, the statistical model’s complexity can make it challenging to interpret the individual contributions of descriptors, thereby limiting practical insights for chemical risk assessment. Finally, this study focuses exclusively on TTR-binding activity and does not evaluate broader biological consequences. Comprehensive assessment of thyroid-axis perturbation requires integration with receptor-mediated, metabolic, and immune-related endpoints.

Although the models demonstrated reasonable predictive performance, the methodological limitations described above affect their predictive reliability and regulatory applicability. The restricted chemical space of the training data may limit the robustness of predictions for structurally novel or underrepresented compounds, and reliance on the specific assay conditions used in the Tox24 challenge may reduce generalizability to alternative experimental platforms. Additionally, while cross-validation and applicability domain analysis mitigate overfitting, they cannot fully eliminate uncertainty when extrapolating beyond the modeled domain. These considerations suggest that the models are best suited for screening-level tool prioritization rather than for definitive regulatory decision-making. Future work incorporating broader chemical coverage and multiple assay sources will be important for advancing regulatory readiness.

To improve the current models, several future steps can be taken. Expanding the dataset to include a broader range of chemicals is crucial, possibly through public databases or new experimental studies. Adding more molecular descriptors that capture dynamic interactions could boost predictive accuracy and enhance the interpretability of how specific molecular descriptors and features influence TTR-binding affinity. Exploring the integration of computational tools that combine different modeling techniques may enhance both accuracy and robustness.

Validating our model predictions through in vitro experiments is essential for establishing biological relevance. Ultimately, the model may serve as a screening-level tool and chemical prioritization within tiered testing or integrated assessment strategies, helping to identify chemicals for further experimental evaluation of thyroid-related activity.

## 4. Conclusions

Our study presents a screening-level, machine learning-based QSAR model guided by OECD guidelines to screen chemicals for potential disruption of the thyroid system, underscoring its significance for toxicology and environmental health. The GBR model outperformed other machine learning models as an in silico screening-level tool, demonstrating its practical applicability. However, the model’s performance indicates room for improvement, particularly on external validation datasets. Our machine learning models, GBR and RFR, yield similar mechanistic insights independently, with ALOGP, SpMax_B(v), PCR, EE_B(s)/VR2_Dz(Z), and GATS1i emerging as key features. This agreement supports the robustness of our models statistically and indicates that they capture patterns consistent with established TTR-binding mechanisms observed across chemical classes and modeling strategies.

By integrating empirical structure–activity evidence with machine learning feature importance, this study demonstrates that hydrophobicity, steric effects, branching, connectivity, and ionization/electronic properties form the mechanistic foundation of TTR-mediated thyroid-axis perturbation. This alignment strengthens confidence in our models’ predictive utility for both environmental risk assessment and drug design. Future improvements may include expanded coverage of chemical space, incorporation of additional mechanistically interpretable descriptors, and integration with complementary thyroid-related endpoints. Within its intended context, the model provides a robust framework for screening-level prioritization of TTR-binding chemicals in support of endocrine hazard assessment.

## Figures and Tables

**Table 1:** Summary statistics of TTR-binding values in the training, test, and validation sets.

Datasets	Count	Mean	Median	Min	Max	Standard Deviation
Test	199	33.44	21.73	–23.54	111.12	33.3
Training	1001	40.97	30.1	–45	111.1	36.46
Validation	299	40.86	30.6	–41.6	110.9	35.84

**Table 2:** Top 25 molecular descriptors names, types, and description details.

Descriptor Name	Type	Description	Functional-Group/Structural Relevance
ALogPS_logS	Solubility descriptor	Predicted solubility in water	Polarity and hydrogen bonding
Mi (Dragon7)	Constitutional indices	Mean first ionization potential	Electronic effects (nitro, halogens)
nBM (Dragon7)	Constitutional descriptor	Number of multiple bonds	Aromaticity and unsaturation
PCR (Dragon7)	Walk and path counts	Ratio of multiple path count over path count	Structural complexity
VE2_Dz(Z)	2D matrix-based descriptor	Barysz eigenvector weighted by atomic number	Heavy atoms and halogens
VR2_Dz(Z)	2D matrix-based descriptor	Randic-like index weighted by atomic number	Atomic composition
SpDiam_B(m)	Topological descriptor	Spectral diameter weighted by atomic mass	Molecular size
EE_B(m)	Topological descriptor	Estrada-like index weighted by atomic mass	Overall complexity
AVS_B(v)	2D matrix-based descriptor	Vertex sum weighted by van der Waals volume	Hydrophobic bulk
SpPosA_B(v)	2D matrix-based descriptor	Spectral positive sum weighted by van der Waals volume	Steric volume
SpMax_B(v)	2D matrix-based descriptor	Leading eigenvalue weighted by van der Waals volume	Bulky scaffolds
SpPosA_B(p)	2D matrix-based descriptor	Spectral positive sum weighted by polarizability	Halogenated aromatics
SpMax_B(p)	2D matrix-based descriptor	Leading eigenvalue weighted by polarizability	Polarizable groups
EE_B(s)	2D matrix-based descriptor	Estrada index weighted by intrinsic state	Electronic heterogeneity
GATS1i	2D autocorrelation descriptor	Geary autocorrelation lag 1 weighted by ionization potential	Local electronic effects
SpMax1_Bh(v)	Burden eigenvalues	Largest eigenvalue weighted by van der Waals volume	Dominant hydrophobic feature
SpMax4_Bh(s)	Burden eigenvalues	Fourth largest eigenvalue weighted by intrinsic state	Substituent effects
SpMin1_Bh(e)	Burden eigenvalues	Smallest eigenvalue weighted by electronegativity	Electronegative atoms
SpMax_EA(bo)	Edge adjacency indices	Leading eigenvalue weighted by bond order	Conjugation
TDB01v	3D autocorrelation descriptor	3D distance lag 1 weighted by van der Waals volume	Short-range sterics
TDB03v	3D autocorrelation descriptor	3D distance lag 3 weighted by van der Waals volume	Long-range sterics
Uc	Molecular properties	Unsaturation count	Aromatic systems
MLOGP	Molecular properties	Moriguchi logP	Hydrophobicity
ALOGP	Molecular properties	Ghose–Crippen logP	Lipophilicity
ALOGP2	Molecular properties	Squared Ghose–Crippen logP	Extreme hydrophobicity

**Table 3:** Performance of machine learning models using training, test, and validation sets. Reported metrics include the coefficient of determination (*R*^2^/*Q*^2^) and root mean square error (RMSE).

Models	Train (n = 1001) *R*^2^	Test (n = 199) *Q*^2^	Validation (n = 299) *Q*^2^	Cross-Validation (Fold = 5) Mean *R*^2^ ^(c)^	Train (n = 1001) RMSE	Test (n = 199) RMSE	Validation (n = 299) RMSE	Cross-Validation (Fold = 5) Mean RMSE ^(c)^
RFR	0.90	0.54	0.55	0.45	11.54	22.51	23.99	26.62
GBR	0.89	0.58	0.55	0.47	12.29	21.47	23.89	26.26
RSVM	0.64	0.45	0.46	0.48	21.97	24.57	26.35	26.57
SVM	0.24	0.31	0.24	0.18	31.74	27.64	31.09	32.76
LR	0.28	0.30	0.25	0.24	30.93	27.88	30.91	31.45
Consensus 1 ^a^	0.90	0.57	0.56	NA	11.59	21.69	23.64	NA
Consensus 2 ^b^	0.90	0.57	0.56	NA	11.61	21.72	23.64	NA

^a^ Using 1/MSE as the weight to combine RFR and GBR predictions as the consensus; ^b^ taking the average of RFR and GBR predictions as the consensus; ^c^ these values are the mean RMSE and *R*^2^ across 30 random seeds; NA: not available.

## Data Availability

The original contributions presented in this study are included in the article. The Python 3.11 and R 4.5.0 source code, along with the dataset used for feature selection, model training, and applicability domain analysis, are publicly available on GitHub commit version 6dd4179 at: https://github.com/HSK-BalaBala/Toxics_TTR_2026.git (accessed on 3 March 2026). Further inquiries can be directed to the corresponding author.

## Supplementary Materials

The following supporting information can be downloaded at: https://github.com/HSK-BalaBala/Toxics_TTR_2026.git (accessed on 3 March 2026).

## References

[1] Tetko I.V. (2024). Tox24 Challenge. Chem. Res. Toxicol..

[2] Makarov D.M., Ksenofontov A.A., Budkov Y.A. (2025). Consensus Modeling for Predicting Chemical Binding to Transthyretin as the Winning Solution of the Tox24 Challenge. Chem. Res. Toxicol..

[3] Pan X., Gu Y., Zhou W., Zhang Y. (2025). Enhancing Transthyretin Binding Affinity Prediction with a Consensus Model: Insights from the Tox24 Challenge. Chem. Res. Toxicol..

[4] Cirino T., Pinto L., Iwan M., Dougha A., Lučić B., Kraljević A., Navoyan Z., Tevosyan A., Yeghiazaryan H., Khondkaryan L. (2025). Consensus Modeling Strategies for Predicting Transthyretin Binding Affinity from Tox24 Challenge Data. Chem. Res. Toxicol..

[5] Richard A.M., Judson R.S., Houck K.A., Grulke C.M., Volarath P., Thillainadarajah I., Yang C., Rathman J., Martin M.T., Wambaugh J.F. (2016). ToxCast Chemical Landscape: Paving the Road to 21st Century Toxicology. Chem. Res. Toxicol..

[6] Zoeller R.T., Brown T.R., Doan L.L., Gore A.C., Skakkebaek N.E., Soto A.M., Woodruff T.J., Vom Saal F.S. (2012). Endocrine-disrupting chemicals and public health protection: A statement of principles from The Endocrine Society. Endocrinology.

[7] Vandenberg L.N. (2021). Endocrine disrupting chemicals: Strategies to protect present and future generations. Expert. Rev. Endocrinol. Metab..

[8] Buoso E., Masi M., Limosani R.V., Oliviero C., Saeed S., Iulini M., Passoni F.C., Racchi M., Corsini E. (2025). Endocrine Disrupting Toxicity of Bisphenol A and Its Analogs: Implications in the Neuro-Immune Milieu. J. Xenobiot..

[9] Buoso E., Masi M., Racchi M., Corsini E. (2020). Endocrine-Disrupting Chemicals’ (EDCs) Effects on Tumour Microenvironment and Cancer Progression: Emerging Contribution of RACK1. Int. J. Mol. Sci..

[10] Masi M., Racchi M., Travelli C., Corsini E., Buoso E. (2021). Molecular Characterization of Membrane Steroid Receptors in Hormone-Sensitive Cancers. Cells.

[11] Buoso E., Kenda M., Masi M., Linciano P., Galbiati V., Racchi M., Dolenc M.S., Corsini E. (2021). Effects of Bisphenols on RACK1 Expression and Their Immunological Implications in THP-1 Cells. Front. Pharmacol..

[12] El Mabrouk N., Iulini M., Maddalon A., Galbiati V., Harizi H., Mastouri M., Corsini E. (2023). In Vitro Effects of Cypermethrin and Glyphosate on LPS-Induced Immune Cell Activation. Life.

[13] Iulini M., Bettinsoli V., Maddalon A., Galbiati V., Janssen A.W.F., Beekmann K., Russo G., Pappalardo F., Fragki S., Paini A. (2025). In vitro approaches to investigate the effect of chemicals on antibody production: The case study of PFASs. Arch. Toxicol..

[14] Maddalon A., Cari L., Iulini M. (2023). Impact of endocrine disruptors on peripheral blood mononuclear cells in vitro: Role of gender. Arch. Toxicol..

[15] Passoni F.C., Iulini M., Galbiati V., Marinovich M., Corsini E. (2025). Disrupting Defenses: Effects of Bisphenol A and Its Analogs on Human Antibody Production In Vitro. Life.

[16] De Luca R. (2021). Thyroid hormones interaction with immune response, inflammation and non-thyroidal illness syndrome. Front. Cell Dev. Biol..

[17] Hampton L.M.T., Finch M.G., Martyniuk C.J., Venables B.J., Jeffries M.K.S. (2021). Developmental thyroid disruption causes long-term impacts on immune cell function and transcriptional responses to pathogen in a small fish model. Sci. Rep..

[18] Wenzek C. (2022). The interplay of thyroid hormones and the immune system—Where we stand and why we need to know about it. Eur. J. Endocrinol..

[19] Jongejan R.M.S., Meima M.E., Visser W.E., Korevaar T.I.M., van den Berg S.A.A., Peeters R.P., de Rijke Y.B. (2022). Binding Characteristics of Thyroid Hormone Distributor Proteins to Thyroid Hormone Metabolites. Thyroid.

[20] Köhrle J. (2018). Thyroid Hormones and Derivatives: Endogenous Thyroid Hormones and Their Targets. Methods Mol. Biol..

[21] Mondal S., Raja K., Schweizer U., Mugesh G. (2016). Chemistry and Biology in the Biosynthesis and Action of Thyroid Hormones. Angew. Chem. Int. Ed. Engl..

[22] Richardson S.J. (2007). Cell and molecular biology of transthyretin and thyroid hormones. Int. Rev. Cytol..

[23] Schreiber G. (2002). The evolutionary and integrative roles of transthyretin in thyroid hormone homeostasis. J. Endocrinol..

[24] He J., Xu J., Zheng M., Pan K., Yang L., Ma L., Wang C., Yu J. (2024). Thyroid dysfunction caused by exposure to environmental endocrine disruptors and the underlying mechanism: A review. Chem. Biol. Interact..

[25] Landers K.A., McKinnon B.D., Li H., Subramaniam V.N., Mortimer R.H., Richard K. (2009). Carrier-mediated thyroid hormone transport into placenta by placental transthyretin. J. Clin. Endocrinol. Metab..

[26] Landers K.A., Mortimer R.H., Richard K. (2013). Transthyretin and the human placenta. Placenta.

[27] Liu C., Ha M., Li L., Yang K. (2014). PCB153 and p,p’-DDE disorder thyroid hormones via thyroglobulin, deiodinase 2, transthyretin, hepatic enzymes and receptors. Environ. Sci. Pollut. Res. Int..

[28] Liu C., Shi Y., Li H., Wang Y., Yang K. (2011). p,p’-DDE disturbs the homeostasis of thyroid hormones via thyroid hormone receptors, transthyretin, and hepatic enzymes. Horm. Metab. Res..

[29] Wang Q., Liu C., Zhang Z. (2016). Transthyretin and Normal Human Pregnancy: Mini Review. Crit. Rev. Eukaryot. Gene Expr..

[30] Bohlen M.L., Jeon H.P., Kim Y.J., Sung B. (2019). In Silico Modeling Method for Computational Aquatic Toxicology of Endocrine Disruptors: A Software-Based Approach Using QSAR Toolbox. J. Vis. Exp..

[31] Devillers J., Marchand-Geneste N., Carpy A., Porcher J.M. (2006). SAR and QSAR modeling of endocrine disruptors. SAR QSAR Environ. Res..

[32] Gini G.C. (2025). QSAR: Using the Past to Study the Present. Methods Mol. Biol..

[33] Heo S., Safder U., Yoo C. (2019). Deep learning driven QSAR model for environmental toxicology: Effects of endocrine disrupting chemicals on human health. Environ. Pollut..

[34] Sakkiah S., Guo W., Pan B., Kusko R., Tong W., Hong H. (2018). Computational prediction models for assessing endocrine disrupting potential of chemicals. J. Environ. Sci. Health C.

[35] Vuorinen A., Odermatt A., Schuster D. (2013). In silico methods in the discovery of endocrine disrupting chemicals. J. Steroid Biochem. Mol. Biol..

[36] Manganelli S., Roncaglioni A., Mansouri K., Judson R.S., Benfenati E., Manganaro A., Ruiz P. (2019). Development, validation and integration of in silico models to identify androgen active chemicals. Chemosphere.

[37] Ruiz P., Sack A., Wampole M., Bobst S., Vracko M. (2017). Integration of in silico methods and computational systems biology to explore endocrine-disrupting chemical binding with nuclear hormone receptors. Chemosphere.

[38] Connelly S., Mortenson D.E., Choi S., Wilson I.A., Powers E.T., Kelly J.W., Johnson S.M. (2017). Semi-quantitative models for identifying potent and selective transthyretin amyloidogenesis inhibitors. Bioorga. Med. Chem. Lett..

[39] Eytcheson S.A., Zosel A.D., Olker J.H., Hornung M.W., Degitz S.J. (2024). Screening the ToxCast Chemical Libraries for Binding to Transthyretin. Chem. Res. Toxicol..

[40] Eytcheson S.A., Tetko I.V. (2025). Which Modern AI Methods Provide Accurate Predictions of Toxicological End Points? Analysis of Tox24 Challenge Results. Chem. Res. Toxicol..

[41] Sushko I., Novotarskyi S., Korner R., Pandey A.K., Rupp M., Teetz W., Brandmaier S., Abdelaziz A., Prokopenko V.V., Tanchuk V.Y. (2011). Online chemical modeling environment (OCHEM): Web platform for data storage, model development and publishing of chemical information. J. Comput. Aided Mol. Des..

[42] Todeschini R., Consonni V., Wiley I. (2009). Molecular Descriptors for Chemoinformatics.

[43] Tetko I.V., Tanchuk V.Y., Kasheva T.N., Villa A.E. (2001). Estimation of aqueous solubility of chemical compounds using E-state indices. J. Chem. Inf. Comput. Sci..

[44] Tetko I.V., Tanchuk V.Y. (2002). Application of associative neural networks for prediction of lipophilicity in ALOGPS 2.1 program. J. Chem. Inf. Comput. Sci..

[45] Tetko I.V., Tanchuk V.Y., Villa A.E. (2001). Prediction of n-octanol/water partition coefficients from PHYSPROP database using artificial neural networks and E-state indices. J. Chem. Inf. Comput. Sci..

[46] Kraskov A., Stogbauer H., Grassberger P. (2004). Estimating mutual information. Phys. Rev. E Stat. Nonlin Soft Matter Phys..

[47] Ross B.C. (2014). Mutual information between discrete and continuous data sets. PLoS ONE.

[48] Kozachenko L. (1987). Sample estimate of the entropy of a random vector. Probl. Pered. Inform..

[49] Gadaleta D., Mangiatordi G.F., Catto M., Carotti A., Nicolotti O. (2016). Applicability domain for QSAR models: Where theory meets reality. Int. J. Quant. Struct. -Prop. Relatsh..

[50] Kutner M.H., Nachtsheim C., Neter J., Li W., Kutner M.H. (2005). Applied Linear Statistical Models.

[51] Organisation de Coopération et de Développement Economiques, Organisation for Economic Co-operation and Development (2014). Guidance Document on the Validation of (Quantitative) Structure-Activity Relationship [(Q)SAR] Models.

[52] Tropsha A. (2010). Best Practices for QSAR Model Development, Validation, and Exploitation. Mol. Inf..

[53] Natekin A., Knoll A. (2013). Gradient boosting machines, a tutorial. Front. Neurorobotics.

[54] Yang X., Lyakurwa F., Xie H., Chen J., Li X., Qiao X., Cai X. (2017). Different binding mechanisms of neutral and anionic poly-/perfluorinated chemicals to human transthyretin revealed by In silico models. Chemosphere.

[55] Yang X., Ou W., Zhao S., Wang L., Chen J., Kusko R., Hong H., Liu H. (2021). Human transthyretin binding affinity of halogenated thiophenols and halogenated phenols: An in vitro and in silico study. Chemosphere.

[56] Evangelista M., Chirico N., Papa E. (2024). In silico models for the screening of human transthyretin disruptors. J. Hazard. Mater..

[57] Evangelista M., Chirico N., Papa E. (2025). New QSAR Models to Predict Human Transthyretin Disruption by Per- and Polyfluoroalkyl Substances (PFAS): Development and Application. Toxics.

[58] Kar S., Sepúlveda M.S., Roy K., Leszczynski J. (2017). Endocrine-disrupting activity of per- and polyfluoroalkyl substances: Exploring combined approaches of ligand and structure based modeling. Chemosphere.

[59] Kovarich S., Papa E., Li J., Gramatica P. (2012). QSAR classification models for the screening of the endocrine-disrupting activity of perfluorinated compounds. SAR QSAR Environ. Res..

[60] Papa E., Kovarich S., Gramatica P. (2013). QSAR prediction of the competitive interaction of emerging halogenated pollutants with human transthyretin. SAR QSAR Environ. Res..

[61] Weiss J.M., Andersson P.L., Lamoree M.H., Leonards P.E., van Leeuwen S.P., Hamers T. (2009). Competitive binding of poly- and perfluorinated compounds to the thyroid hormone transport protein transthyretin. Toxicol. Sci..

[62] Harju M., Hamers T., Kamstra J.H., Sonneveld E., Boon J.P., Tysklind M., Andersson P.L. (2007). Quantitative structure-activity relationship modeling on in vitro endocrine effects and metabolic stability involving 26 selected brominated flame retardants. Environ. Toxicol. Chem..

[63] Yang W., Shen S., Mu L., Yu H. (2011). Structure-activity relationship study on the binding of PBDEs with thyroxine transport proteins. Environ. Toxicol. Chem..

[64] Rybacka A., Rudén C., Tetko I.V., Andersson P.L. (2015). Identifying potential endocrine disruptors among industrial chemicals and their metabolites—Development and evaluation of in silico tools. Chemosphere.

[65] Zhang J., Kamstra J.H., Ghorbanzadeh M., Weiss J.M., Hamers T., Andersson P.L. (2015). In Silico Approach To Identify Potential Thyroid Hormone Disruptors among Currently Known Dust Contaminants and Their Metabolites. Environ. Sci. Technol..

[66] Charest N., Sinclair G., Eytcheson S.A., Chang D.T., Martin T.M., Lowe C.N., Paul Friedman K., Williams A.J. (2025). Combined In Vitro and In Silico Workflow to Deliver Robust, Transparent, and Contextually Rigorous Models of Bioactivity. J. Chem. Inf. Model..

[67] Natesan S., Balaz S. (2013). Rigorous incorporation of tautomers, ionization species, and different binding modes into ligand-based and receptor-based 3D-QSAR methods. Curr. Pharm. Des..

[68] Natesan S., Wang T., Lukacova V., Bartus V., Khandelwal A., Balaz S. (2011). Rigorous treatment of multispecies multimode ligand-receptor interactions in 3D-QSAR: CoMFA analysis of thyroxine analogs binding to transthyretin. J. Chem. Inf. Model..

[69] Ren X.M., Guo L.-H. (2012). Assessment of the Binding of Hydroxylated Polybrominated Diphenyl Ethers to Thyroid Hormone Transport Proteins Using a Site-Specific Fluorescence Probe. Environ. Sci. Technol..

[70] Judson R.S., Houck K.A., Kavlock R.J., Knudsen T.B., Martin M.T., Mortensen H.M., Reif D.M., Rotroff D.M., Shah I., Richard A.M. (2010). In vitro screening of environmental chemicals for targeted testing prioritization: The ToxCast project. Environ. Health Perspect..

[71] Gore A.C., Chappell V.A., Fenton S.E., Flaws J.A., Nadal A., Prins G.S., Toppari J., Zoeller R.T. (2015). EDC-2: The Endocrine Society’s Second Scientific Statement on Endocrine-Disrupting Chemicals. Endocr. Rev..

[72] Zoeller R.T., Vandenberg L.N., Turgeon J.L. (2021). Chapter Ten—Endocrine disrupting chemicals and thyroid hormone action. Advances in Pharmacology.

[73] Ankley G.T., Bennett R.S., Erickson R.J., Hoff D.J., Hornung M.W., Johnson R.D., Mount D.R., Nichols J.W., Russom C.L., Schmieder P.K. (2009). Adverse outcome pathways: A conceptual framework to support ecotoxicology research and risk assessment. Environ. Toxicol. Chem..

[74] Yang X., Xie H., Chen J., Li X. (2013). Anionic phenolic compounds bind stronger with transthyretin than their neutral forms: Nonnegligible mechanisms in virtual screening of endocrine disrupting chemicals. Chem. Res. Toxicol..

